# Can microfluidics address biomanufacturing challenges in drug/gene/cell therapies?

**DOI:** 10.1093/rb/rbw009

**Published:** 2016-03-08

**Authors:** Hon Fai Chan, Siying Ma, Kam W. Leong

**Affiliations:** Department of Biomedical Engineering, Department of Systems Biology, Columbia University, New York, NY 10032, USA

**Keywords:** microfluidics, biomanufacturing, nanoparticle, microencapsulation, microfiber

## Abstract

Translation of any inventions into products requires manufacturing. Development of drug/gene/cell delivery systems will eventually face manufacturing challenges, which require the establishment of standardized processes to produce biologically-relevant products of high quality without incurring prohibitive cost. Microfluidicu technologies present many advantages to improve the quality of drug/gene/cell delivery systems. They also offer the benefits of automation. What remains unclear is whether they can meet the scale-up requirement. In this perspective, we discuss the advantages of microfluidic-assisted synthesis of nanoscale drug/gene delivery systems, formation of microscale drug/cell-encapsulated particles, generation of genetically engineered cells and fabrication of macroscale drug/cell-loaded micro-/nano-fibers. We also highlight the scale-up challenges one would face in adopting microfluidic technologies for the manufacturing of these therapeutic delivery systems.

One aspect of biomanufacturing is the use of technology to fabricate biologically relevant materials and devices wherein biological components and/or processes are included. In development of pharmaceutical and medicinal products, biomanufacturing represents one critical step in translating the process performed in academic laboratories into commercial-scale manufacturing. In cell-based therapeutics e.g. the successful cases of product approval by the Food and Drug Administration (FDA) and subsequent commercialization are vastly out-numbered by prevalent failures of product development, which can be partly attributed to high cost of products and technical hurdles encountered when the manufacture process is scaled up [1]. Currently, the laboratory-scale preparation of human cells or tissues is a highly specialized activity that is subjected to user-to-user variation. Automation ought to be introduced for standardizing procedures and achieving flexibility in production to adapt to potential market changes.

Meanwhile, biomanufacturing plays a significant role in commercializing delivery systems for drug and gene therapies that are predominantly in micro-/nano-particulate form. Since the first FDA approval of drug delivery system (DDS), Lupron Depot, in 1989, more than 30 DDS are now commercially available to treat a wide range of diseases (Fig. 1). In contrast, the commercialization of gene therapy has stalled [2]. The first commercialized gene therapy, Glybera (approved in Europe only in 2013), leverages on viral vector to deliver the target gene and is expected to cost >$1 million/treatment [3]. Since viral vectors are associated with toxicity, immunogenicity and high cost, development of gene delivery systems using non-viral vector has continued to gain momentum as demonstrated by the steady increase of research articles published on the topic [2]. In general, the low transfection efficiency is an obstacle of non-viral gene delivery [4]. In addition to material composition, fabrication methods have been shown to affect the transfection capability of non-viral gene vector [5]. Moreover, *in vitro* and *in vivo* properties of drug/gene delivery systems depend on a number of characteristics such as size, surface charge, and drug/gene loading efficiency that are in turn controlled by fabrication methods [6].

**Figure 1.**
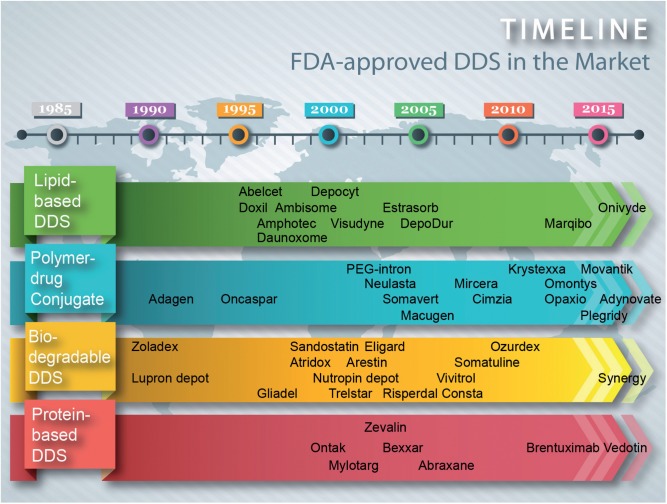
Timeline showing some examples of DDS approved by FDA

The current Good Manufacturing Practices (cGMP) for biomanufacturing issued by FDA require standardized manufacturing processes to be established to ensure products (e.g. drugs) possess the desired characteristics in terms of identity, strength, quality and purity [7]. Microfluidics, the manipulation of fluid flow in small scale (nano- or pico-liter), has been studied for fabricating biologically relevant materials owing to the multiple advantages it offers. Here, we review the rationale and examples of adopting microfluidics for fabrication or formulation in the fields of/drug/gene/cell therapies, and highlight how microfluidics may address existing and future biomanufacturing challenges.

## Introduction of microfluidic technologies for biofabrication

Microfluidics is a rapidly evolving field with applications encompassing diagnostics, molecular biology, high-throughput screening and material fabrication [8]. The basic components of microfluidic technology comprise a device with one or more channels that are <1 mm in dimension and a pump such as syringe or peristaltic pump to drive liquid flow [9]. The general benefits of microfluidic platform include but not limited to miniaturization, reduced reagent consumption, rapid heat and mass transfer due to high surface-to-volume ratio and enhanced processing accuracy and efficiency in predictable liquid flow at small scales [10]. Conventional macroscale bioprocessing can be reproduced in microfluidic device with minimal reagent input and device footprint, leading to reduced cost and better controllability. In particular, emulsion droplets are produced when two or more immiscible streams, supplemented with a surfactant, are introduced in microfluidic platform concurrently [11, 12]. These emulsion droplets compartmentalize the bulk reaction mixture into isolated, uniform-sized reactors. This results in consistent material fabrication and prevents cross-contamination of reagents across droplets.

**Figure 2.**
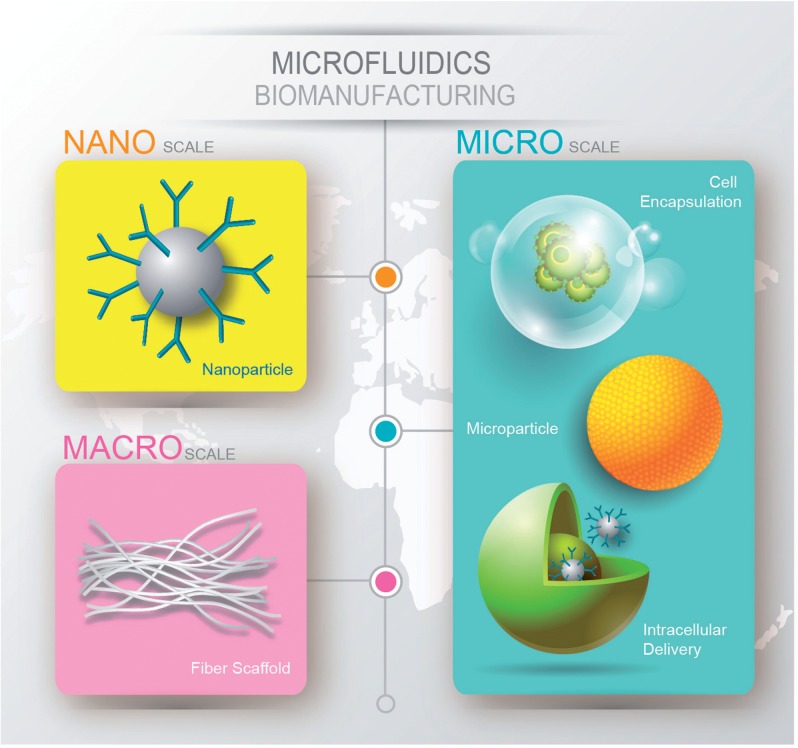
Illustration of nano- to macro-scale products manufactured with microfluidics

Examples of adopting microfluidic technologies for biomanufacturing in the fields of cell/drug/gene therapies can be categorized by the length scale of products, ranging from nano- to macro-scale (Fig. 2). Within the category of nanoscale products, nanoparticles (NPs) loaded with drugs through physical encapsulation, adsorption or covalent conjugation and nanocomplexes (NC) carrying nucleic acid through electrostatic binding can be synthesized in microfluidic platform [13]. At the microscale, microparticles can be produced for controlled drug delivery [14]. In a variation of the theme, cells can be transfected or loaded with macromolecules in microfluidic platform to secrete therapeutic products for cell-mediated drug delivery [15]. Alternatively, cells can be encapsulated in hydrogels within the droplets for immunoprotection in artificial organ applications [16]. At the macroscale, scaffolds of microfluidic-generated microfibers can be applied as a patch for controlled drug/gene delivery or implantable engineered tissue [17, 18]. We will examine each category and discuss the advantages of microfluidics-mediated fabrication followed by the challenges of biomanufacturing.

## Microfluidic synthesis of nanoscale Drug/gene delivery system

**Figure 3.**
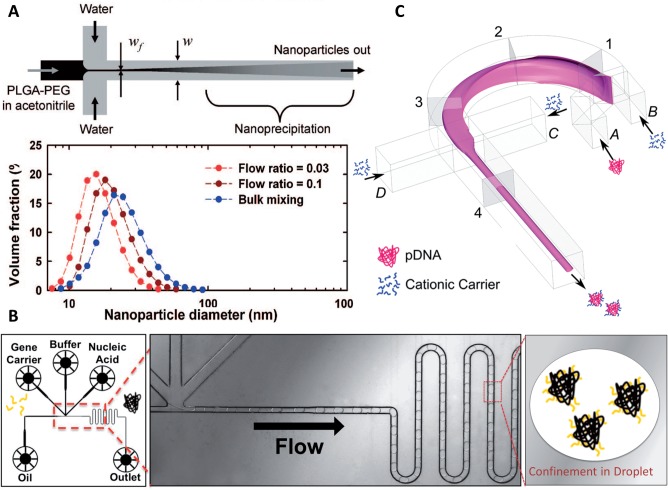
**(A)** Top: illustration of nanoprecipitation performed in hydrodynamic flow-focusing channel where solvent exchange occurs via rapid diffusion along the interface of two phases. Bottom: the size distribution of NP generated by different approaches (flow ratio = 0.03 and 1 refer to ratio of flow rates in microfluidic fabrication) (reprinted (adapted) with permission from [23]. Copyright (2008) American Chemical Society). **(B)** Illustration of microfluidics-assisted assembly of NC in picoliter droplets. Plasmid DNA, buffer, gene carrier and oil are introduced into each channel with syringe pumps. The DNA and gene carrier are then confined into individual droplets and subsequently self-assembled through electrostatic interaction (reprinted (adapted) with permission from [36]. Copyright (2011) American Chemical Society). **(C)** Schematic of the 3D-hydrodynamic flow focusing for NC synthesis. The DNA solution is injected through inlet A, while the polymer solution is injected from inlets B–D (reprinted (adapted) with permission from [41]. Copyright (2011) American Chemical Society).

Nanoscale DDS holds tremendous promise for disease treatment since it can encapsulate poorly soluble drugs and release them in a controlled manner, protect therapeutic molecules from premature excretion or immune reaction and be chemically modified for targeting specific disease tissues [19]. Examples of commercially available nanoscale DDS include liposome-based (e.g. Doxil) and protein-based NP (e.g. Abraxane). Despite the advantages associated with nanoscale DDS and decades of research, translation of NP to the clinic has been slow compared with small-molecule drugs [13]. One critical barrier is the difficulty of synthesizing NPs with tunable physicochemical properties and minimal batch-to-batch variations and in sufficient quantity for clinical applications [20]. Synthetic protocol dictates the size of a NP and its subsequent biodistribution, one of the most important parameters that determines the efficacy of a NP delivery system. NP <20 nm will be removed from circulation via the reticuloendothelial system within a few hours, whereas larger ones will be trapped in the liver and the spleen within minutes [21]. Polymeric micelles with a diameter of 30 nm only (but not with a diameter of 50, 70 or 100 nm) could effectively penetrate poorly permeable pancreatic tumors [22]. Traditional bulk mixing such as nanoprecipitation and emulsification-based methods rely on self-assembly of precursor molecules when there is a change in solvent quality. NP synthesized by bulk mixing are prone to polydispersity, large particle size (often >150 nm in the case of emulsification-solvent evaporation) and batch-to-batch variations [23]. In bulk mixing, longer timescale of solvent exchange (in the order of seconds) than that of precursors to nucleate and grow results in undesired NP aggregation [13]. Using microfluidic device, nanoprecipitation can be conducted in hydrodynamic flow focusing, where the precursor solution is focused into a thin stream between two streams of anti-solvent and rapid solvent exchange occurs via diffusion through the interface (Fig. 3A) [24]. The short mixing time (in the order of microseconds) yields smaller NP (<100 nm) with more uniform size [23]. A microfluidic device capable of focusing stream hydrodynamically in 3D could reduce the particle size further since NP aggregation on channel wall was prevented [25]. Nevertheless, the nanoprecipitation approach requires the utilization of two miscible solvents (e.g. acetone and water) in which the drug and polymer dissolve in one but not the other, thereby introducing variation in drug encapsulation efficiency (EE) depending on the nature of the drug, polymer and solvent [26, 27]. In the case of fabricating docetaxel-loaded PLGA NP, the EE was not high in both microfluidic synthesis (51%) and bulk mixing (45%) because docetaxel precipitated at a different solvent condition than the polymer [23]. Entrapping hydrophilic drug is especially challenging for nanoprecipitation [26]. On the other hand, the emulsification-based approach has been shown to better encapsulate hydrophilic drugs and protein due to the shielding effect of disperse phase [28, 29]. One study reported the entrapment of hydrophilic drug in NP using a microfluidic device to generate water-in-oil-in-water (w/o/w) emulsion with significantly higher EE (97%) than that obtained by bulk mixing (57%) [30]. The authors attributed reduced drug loss to fewer steps involved in microfluidic-assisted emulsion production compared with sequential bulk mixing of multiple phases. The size uniformity of NP also improved; however, a particle size limit of >70 nm was observed [31]. Future development of generating nanoemulsion in micro- or nano-fluidic device is expected to contribute to synthesizing NP of smaller size, better uniformity and higher EE.

The expansion of potential genetic targets for intervention of inherited and acquired diseases has fueled the development of gene therapy. The therapeutic potential of gene therapy depends largely on effective intracellular delivery systems [32]. As a safer and more cost-effective alternative to viral gene delivery, non-viral gene delivery often requires the use of cationic polymers or lipids to condense negatively charged nucleic acids (e.g. plasmid DNA, mRNA, siRNA) into nano-sized polyplex and lipoplex, respectively. Innovations in carrier design have given rise to sophisticated delivery systems [33]. Nevertheless, issues such as low transfection efficiency and toxicity of unreacted cationic molecules render non-viral gene delivery prohibitively inefficient for clinical translation [34]. In addition to carrier material composition, the process of NC production assumes an important role in optimizing the physicochemical attributes of NC [35, 36]. The assembly of NC by charge neutralization is a highly energetic process that occurs in milliseconds [37, 38]. Bulk preparation by pipetting, or vortex mixing introduces great variability into the quality of the NC formed, leading to poor biological reproducibility [39]. The difficulty of manufacturing NC in a controlled, reproducible and scalable manner also hinders their clinical translation. Similar to the case of nanoscale DDS, emulsion-based approach and hydrodynamic flow focusing in microfluidics have been shown to improve the quality of NC produced [35, 37, 40, 41]. Employing water-in-oil emulsion droplets, approximately same amount of reagents are encapsulated in each droplet. Confined diffusion within droplets and rapid mixing as the droplets move along the channel facilitate charge neutralization between oppositely charged molecules [36]. The resulting NC displayed smaller and more uniform size, lower surface charge (lower zeta-potential), better stability, higher transfection efficiency and lower cytotoxicity than NC created by bulk mixing (Fig. 3B). A quantum dot-Förster resonance energy transfer assay revealed slower unpacking of microfluidic-generated NC to release its payload intracellularly, which might result in higher chances of nucleic acids penetrating the nucleus [21]. In hydrodynamic flow focusing, nucleic acid stream is focused by streams of cationic lipids and polymers into a narrow stream where rapid mixing occurs through diffusion across the interface in microseconds [42]. The resulting NC again were smaller and more monodispersed, transfected cells better without inducing higher toxicity than the bulk mixed counterparts. Furthermore, to prevent aggregation of NC on channel wall and enhance the vertical diffusion, a ‘microfluidic drifting’ technique was developed to achieve 3D hydrodynamic focusing in a single-layered microfluidic device (Fig. 3C) [41]. Favorable attributes were exhibited by the NC produced and they were further enhanced when acoustic perturbation was applied.

In addition to particle size, particle shape has been shown to affect cellular uptake and *in vivo* transport of NP and NC, with rod- or worm-like structure exhibiting superior circulation profile and cellular uptake over spherical particles [43, 44]. It is challenging to fabricate non-spherical drug-loaded particles with traditional mixing procedures. To address the issue, a top-down lithographic fabrication method called PRINT (Particle Replication In Non-wetting Templates) was developed to fabricate micro- and nano-particle of defined shapes [45]. A non-wetting elastomeric mold containing cavities of predefined shapes is used to contain precursor solution for gelling or crosslinking that allows high-throughput production of NP. In contrast to the static production of PRINT, continuous flow lithography combines the advantages of photolithography and microfluidics to continuously form morphologically complex particles [46]. Precursor solution flows along a microfluidic channel underneath which a photomask with defined shapes is placed and pulses of UV light are applied. Particles of defined shapes are formed and flushed to the outlet for collection. This technology has the potential to be scaled up for mass production of NP but is currently limited to photocrosslinking reaction. An improved version of the technology is called stop-flow lithography, where fluid flow is stopped during polymerization to boost the resolution of particles form [47]. Recently, it was discovered that the shape of micellar polyplex could be tuned by controlling the solvent polarity during particle formation [48]. According to the report, a higher degree of uniformity of various polyplex structures was obtained by titrating solvent polarity after the polyplex was prepared than bulk mixing the reagents under the same solvent condition. Since bulk mixing introduces variability into polyplex condensation, the use of microfluidic platform such as emulsion droplet or hydrodynamic focusing may circumvent the problem and provide a more controllable environment for direct synthesis of polyplex of defined shape.

After discussing the potential of microfluidic platform to achieve reproducible fabrication of nanoscale drug/gene delivery system, we now examine the throughput and scalability of microfluidic platform. The example of NP dose ranges from 50 to 500 mg/human for Doxil and Abraxane in each administration. This would require a multi-kilogram manufacturing process operating under cGMP to meet the production requirement. For hydrodynamic flow focusing, the early design leveraged on diffusive mixing between the focused and surrounding streams that occurred only at low flow rate (i.e. low Reynolds number), which gave a productivity of NP at 0.003 g/h [23]. Subsequent designs introduced convective and microvortex mixing in high speed flow that increased the productivity to 0.005 and 3 g/h, respectively [49, 50]. The vortex and turbulence seen in high speed flow would enable even shorter mixing time and formation of smaller NP. A coaxial turbulent jet mixer could operate at a Reynolds number of above 3500 that resulted in a production rate of 130 g/h [51]. Another study demonstrated the incorporation and operation of multiple flow focusing channels on a same device that enhanced the throughput tremendously and proved the scalability of the technology [52]. Achieving sufficient productivity for clinical application is one target. Developing a high-throughput platform for rapid, combinatorial synthesis and optimization of NP also receives considerable attention. A microfluidic flow focusing device with multiple inlets was described that could mix different NP precursors prior to NP synthesis for screening [53]. In the emulsion-based approach, the disperse phase flow rate used to generate poly(lactic-co-glycolic acid) NP was ∼32 μg/ml versus ∼50 mg/ml in the hydrodynamic microvortexing approach [50, 54]. For NC synthesis, the typical working flow rate of nucleic acid and carrier combined for emulsion formation was ∼7.5 μl/min compared with ∼60 μl/min in the case of hydrodynamic flow focusing [27, 55]. Increasing flow rate during emulsion formation is tricky as variation of the flow conditions can lead to transition between stable droplet production and occurrence of jetting [56]. Nevertheless, it is feasible to increase throughput by running multiple droplet generators in parallel, such as utilizing a microfluidic module containing 128 cross-junctions that can produce droplets at a rate of 5.3 ml/min [57]. Overall, concerted efforts have been made to verify the potential of microfluidics to advance nanoscale drug/gene delivery system production and future work should focus on improving drug encapsulating efficiency and fabricating particles of defined shape.

## Encapsulation of cell/drug in microfluidic-generated microparticle/microgel for delivery of therapeutic products

Degradable microparticles/microspheres have been widely used as matrices for drug delivery [58], in which encapsulated drug is released by diffusion through the matrix or erosion of the matrix itself [59]. One example is Lupron Depot, a FDA-approved drug-loaded microsphere intended for controlled drug release after intramuscular injection. Particle size is one important determinant of drug release profile [60]. Traditional procedures of fabricating microparticles are based on droplet formation via sonication and mechanical homogenization followed by solidification of particles (e.g. solvent evaporation, polymerization) [61], which result in size polydispersity and necessitate further filtration step to modulate particle size distribution.

**Figure 4.**
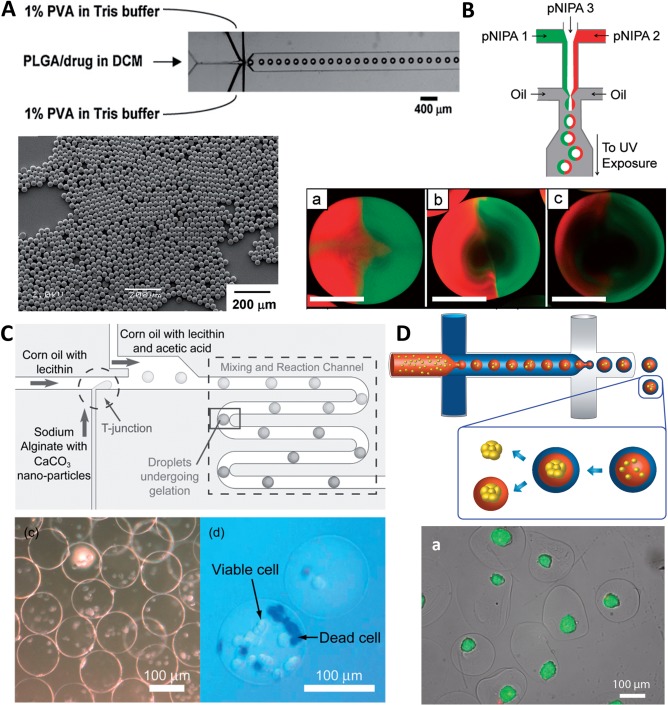
**(A)** Top: optical microscopy image showing the flow-focusing device used to generate microparticles. Bottom: SEM image of monodisperse PLGA microparticles generated in microfluidics (reprinted from [14]. Copyright (2009), with permission from John Wiley and Sons). **(B)** Top: schematic of formation of janus particle in a microfluidic device with three inlets. Bottom: varying the flow rates of the two outer polymer phases, the untagged center polymer phase, and the emulsifying oil phase yields particles with different inner morphology (reprinted (adapted) with permission from [71]. Copyright (2010) American Chemical Society). **(C)** Top: schematic view of alginate hydrogel microbeads production in a T-junction type microfluidic device. Droplets of Na-alginate containing CaCO_3_ NPs are formed at the T-junction. A stream of “acidic oil” merges with the mainstream and induces Ca2+ release by reducing pH for alginate gelation. Bottom: bright field and live-dead images of cell-encapsulated alginate microbeads (reprinted from [16], Copyright (2009), with permission from John Wiley and Sons). **(D)** Top: schematic diagram showing double-emulsion droplets are generated for spheroid production. The spheroid can then be encapsulated in microgel after oil shell removal. Bottom: (a) Live/dead staining of spheroids encapsulated in alginate microgel (adapted from [95]).

Microfluidic platform offers a unique advantage in generating uniform-sized emulsion droplet, with tunable size ranging from a few to hundreds of microns. Homogeneity can be seen in particle size as well as drug distribution inside the particle, leading to more sustained drug release and the possibility of injecting larger particles since the chance of clogging a needle by the large size fraction is reduced (Fig. 4A) [14]. Bypassing the filtration step also increases the overall yield of production. Moreover, the microfluidic platform, especially that made of glass, is compatible with various chemical compounds and therefore can be adapted for the synthesis of different smart drug particles including temperature-, stimulus- and pH-responsive microparticles for triggered drug release [62–64]. The controlled generation of emulsion droplets also facilitates the production of designer microparticles that are impossible to be constructed before. For example, uniform-sized double-emulsion of w/o/w or oil-in-water-in-oil (o/w/o) droplets can be formed via two emulsification steps in one or two microfluidic devices [65, 66]. They can serve as template to produce core-shell microparticles with two different drugs encapsulated in distinct compartments for sequential drug release or the shell modulating the rate of drug release from the core [67–69]. Biphasic, also referred to as Janus, or multi-phasic microparticles can be made by emulsifying two or more parallel-flowing streams of disperse phase and subsequently solidifying the multiphasic droplets (Fig. 4B) [70, 71]. The benefits of such a structure are that drugs encapsulated in two hemispheres can be released simultaneously so they can be of different nature (e.g. hydrophilic and hydrophobic) [72]. Using microfluidic platform, the microparticles can be created with shapes such as sphere, circular disk and rod although the influence of particle shape on drug diffusion properties needs to be determined [73]. In regard to drug encapsulation, the presence of immiscible phase surrounding the emulsion droplets prevents drug loss leading to higher drug EE (>75%) than that achieved with conventional extrusion (40–60%) [74, 75]. Nevertheless, as in the case of NP formation, the emulsion-based approach is hampered with relatively low throughput (∼300 mg/h for single-channel device adopting a disperse phase flow rate of 2 ml/h) [14]. Incorporating multiple (e.g. 15 and 128) droplet generators in 2D or 3D array is possible for single or double-emulsion manufacturing which could significantly increase the overall disperse flow rate to 24–320 ml/h [57, 76].

Immobilizing cells in biocompatible hydrogels offers many attractive features for tissue engineering, such as providing support for anchorage-dependent cells and presenting biochemical cues to module cell behavior [77]. In particular, microencapsulated cells that express therapeutic proteins or growth factors can be transplanted for sustained delivery of therapeutic products *in vivo* [78]. The hydrogel layer can serve as immunoisolation barrier to allow transplantation of foreign cells, such as animal cells or genetically modified cell lines. For effective cell culture and delivery, a few obstacles related to the microencapsulation process need to be overcome. First, conventional microcapsule/microgel formulations rely on droplet extrusion from a nozzle or needle and create large hydrogel (500–1000 μm) [79]. A small gel size is preferred to ensure short diffusion distance and high surface-to-volume ratio for rapid exchange of nutrients and waste. Second, existing problem of size polydispersity results in differential profile of oxygen and nutrients diffusion of each gel and thus difficulty of predicting overall cell survival [80]. Finally, deformed microgels are formed during droplet dripping which might cause fibrotic overgrowth on surround tissue after implantation [81].

To address the challenges, microfluidic-generated emulsion droplet (usually <500 μm) provides a promising alternative for encapsulating cells in equal-sized compartments before the droplet phase is polymerized to produce uniform-sized, cell-laden, spherical microgel [16]. The polymerization of alginate inside droplets has been studied extensively and is carried out through external and internal calcium ion-triggered mechanisms [82]. External gelation is conducted by delivering the cell-containing alginate droplets to a reservoir containing calcium ions that diffuse into the droplets [83]. For the internal gelation, alginate droplets containing insoluble calcium salts (e.g. calcium carbonate) are generated (Fig. 4C) [16, 84]. The continuous phase is then acidified to promote the release of calcium ions from the insoluble salts. A few other biomaterials encapsulated inside droplets can be polymerized externally via applying heat (e.g. collagen), cooling (e.g. agarose, gelatin) and UV light (e.g. poly(ethylene glycol) diacrylate) etc [85–89]. One critical challenge of the microfluidic-assisted biomanufacturing process is to preserve cell viability during droplet formation, polymerization of droplet phase and finally oil phase removal. The cell viability immediately after droplet formation was reported to be over 80% although the presence of immiscible oil phase impeded nutrient replenishment and hence a gradual drop of cell viability inside the droplet over time was observed [90, 91]. For polymerization, mild conditions like transient temperature variation and UV exposure were compatible with cell culture. However, triggering calcium release from insoluble calcium salt by lowing pH could be detrimental after prolonged exposure to acid (e.g. acetic acid), thus alternative method using slow hydrolyzing acid was reported [92]. In some cases, on-chip exchange of acid to another organic phase was necessary to enhance cell survival [93]. Finally, the immiscible phase was typically removed by centrifugation of the microgels suspended in a mixture of culture medium and an oil phase highly immiscible with water. The choice of oil could significantly affect the viability of cells since any residual organic solvent left on microgel surface could be harmful to cells [16]. The centrifugation process could also lead to collapse of microgels or exert excessive mechanical force on the cells that led to reduction in cell survival [85]. Although the immediate cell viability after organic phase removal was reported to be >74%, a number of studies demonstrated a gradual decrease in cell viability or proliferation rate after microgels were extracted and cultured [84, 85], suggesting cell quality could be compromised during the microgel formation and extraction process. An on-chip microgel extraction process was reported to circumvent the centrifugation step to improve cell viability and proliferation [85, 94]. Microgel formation based on double-emulsion droplet generation was also an alternative to avoid the use of hazardous organic phase and centrifugation (Fig. 4D) [95, 96]. Overall, improving the microgel formation process for preserving cell viability and expanding the scope of hydrogel materials used are imperative to the successful translation of the technology.

## Intracellular delivery of macromolecules using microfluidics

Genetically modified cell lines can serve as depot for sustained secretion of therapeutic products (such as factor VIII and IX for treating hemophilia A and B, respectively) [97, 98]. Primary cells such as dendritic cells can be transfected to present antigen for inducing cancer immunity [99]. In addition, stem cells such as mesenchymal stem cells can be genetically modified to overexpress therapeutic proteins to increase their survivability and migration in cell therapy, as well as loaded with non-peptidic drugs or magnetic NPs for enhanced efficacy and externally regulated targeting [100]. The challenge of the approach is to achieve sufficient efficiency of intracellular delivery, especially for some hard-to-transfect cell types including lymphoma cells and embryonic stem cells. In earlier section, we have covered the formulation of NC for non-viral gene delivery by applying microfluidics. Although NC is efficient in nucleic acid delivery, they are in general inefficient for the delivery of proteins.

**Figure 5.**
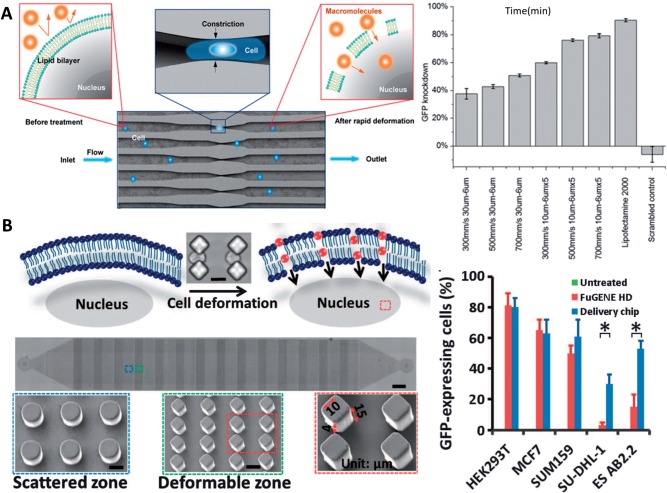
**(A)** Left: illustration of intracellular delivery mechanism whereby the microfluidic constriction generates transient membrane holes on cells when they are deformed. Right: siRNA delivery promotes gene knockdown in live destabilized GFP-expressing HeLa cells, the extent of which depends on device type and cell speed. Lipofectamine 2000 was used as a positive control (adapted from [15], Copyright by the National Academy of Sciences). **(B)** Left: illustration of delivery mechanism and microscopic image of the device structure in which transient membrane holes are generated when cells pass through the microconstriction between the diamond arrays. Right: efficiency of delivery of plasmids encoding GFP in different cell lines [102] (Copyright 2015, the authors, AAAS)

Different microfluidic platforms have been developed with an aim of conducting *in situ* transfection or intracellular delivery at higher efficiency than using conventional methods (e.g. NP-mediated transfection and electroporation) in normal cell culture [15, 95, 101, 102]. For example, water-in-oil droplet was used to encapsulate cells and transfection reagent in order to increase the probability of interaction between them due to confinement effect [90]. Although improved transfection efficiency compared with transfection conducted in culture plate was not observed, higher transfection efficiency was noted in small droplets than in large droplets, indicating the likely effect of microscale confinement. Further development of the technology is required for it to be applicable in routine transfection operation. By forcing cells to flow through a constriction in microfluidic channel, transient holes in membrane were generated to facilitate intracellular delivery of nanomaterial, protein and nucleic acid while maintaining excellent viability (>80%) (Fig. 5A) [15]. The technique was more effective in delivering transcription factors intracellularly than electroporation and transfecting lymphoma cells and mouse embryonic stem cells than using commercial reagents (Fig. 5B). Genome editing was also achieved by deforming cells in the microfluidic channel for single-guided RNA and Cas9 protein penetration without requiring any gene vector [102]. Most importantly, the throughput of the technology is very high, reaching a rate of 20 000 cells/s [15]. Given the potential of scaling up by incorporating multiple channels or operating multiple devices simultaneously, this technology should play a vital role in advancing intracellular delivery for cell and drug therapies in the future.

## Microfluidic-generated micro-/nano-fibers as macroscale cell/DDS

Scaffolds composed of micro- and nano-scale fibers hold great promise as macroscale cell/DDS. The small diameter of fibers provides short diffusion distance and high surface-to-volume ratio for mass exchange and drug release, making the fibers favorable cell culture platform and localized drug delivery vehicle [103]. The porous structure enables cell ingrowth to facilitate tissue regeneration and drug uptake by cells. A range of methods have been reported for manufacturing fibers [104]. Melt spinning begins with heating polymer above its melting point before extruding it through a spinneret. The high temperature (>150°C) required demands the use of expensive equipment and prevents the encapsulation of cell and protein inside the fibers. Wet spinning, which forms fibers by injecting a pre-polymer solution into a coagulation bath for polymerization to occur, faces possible limitation of prolonged exposure of harmful chemical in the bath for cell and protein encapsulation. Electrospinning, which has been intensively studied in the past decade, can effectively fabricate nanoscale fibers of dimension comparable to native extracellular matrix, hence can be used to construct a biomimetic scaffold to direct cellular behavior. The disadvantage lies in the use of high voltage to draw the charged solution that precludes the loading of sensitive biological materials. Moreover, dehydration and fiber stretching during fiber formation contribute to significant death of cell encapsulated [105]. For drug loading, large discrepancies in level of loading efficiency were reported, with one study claiming the EE was 0.003% whereas two others reporting values of 41% and >90%, respectively [18, 106, 107]. The difference in charge densities between the protein and polymer solutions was suggested to be the cause of inefficient encapsulation.

**Figure 6.**
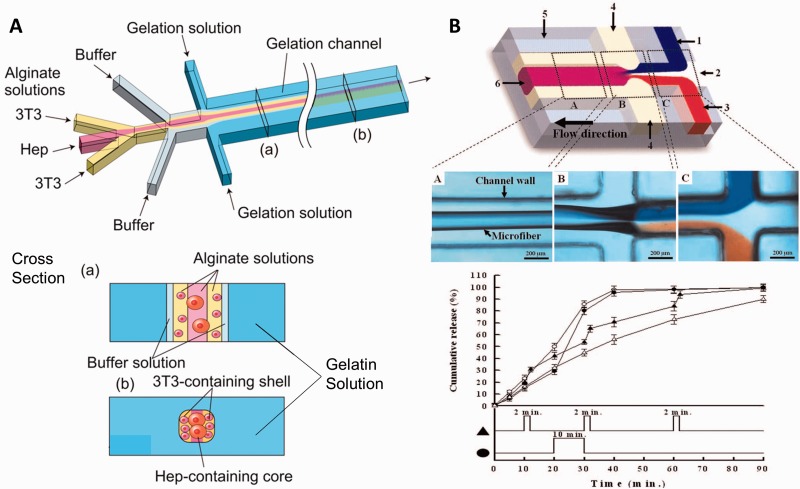
**(A)** Top: microfluidic system for fabricating alginate hydrogel microfibers containing hepatocytes and 3T3 cells. Bottom: illustrations correspond to cross-sectional images at (a) and (b) in the upper image (reprinted from [109], Copyright (2012), with permission from Elsevier). **(B)** Top: the diagram of the microfluidic system and fabrication of alginate microfiber loaded with drug and magnetic iron oxide NPs for triggered drug release. 1, CaCl_2_ solution; 2, deionized water; 3, solution of alginate, drug and iron oxide; 4, oil. Below are photographs of observation positions. Bottom: release profiles of drug from microfibers without magnetic stimulation as the control (empty triangle), with 2-min stimulation at the 10th, 30th and 60th minute (filled triangle), with a 10-min stimulation after the 20th minutes (filled circle) and with a continuous stimulation from the beginning (empty circle) (adapted from [113])

Using microfluidics, coaxial flow of a pre-polymer and a cross-linking agent in flow focusing channel resulted in continuous production of fibers [17]. The typical diameters of the fabricated fibers are between ten to several hundreds of micrometers; however, one study leveraged on dehydration of polymer stream inside the channel to produce nanoscale fiber (>70 nm in width) [108]. Because the polymerization reaction occurs in a hydrated environment and the cross-linking agent can be rapidly diluted or removed by transferring the fibers into a buffer bath, excellent viability of cell encapsulated (>80%) was reported (Fig. 6A) [109–111]. Furthermore, the controlled polymerization inside the microfluidic channel reduces drug loss during encapsulation, with EE reported to be 58–90% (Fig. 6B) [112, 113]. The flexibility of microfabricated platform design also allows the generation of fibers of various structures, such as fibers coded with varying chemical composition and topography for spatially controlled co-culture of encapsulated cells and controlled presentation of topographical cues for cells cultured on the fibers, respectively [114]. Nevertheless, the drawback of this technology is that the flow rate of the pre-polymer solution used is typically low (several μl/min compared with several ml/min in the case of electrospinning) [18, 112]. The low flow rate is important to maintain small diameter of the fiber and to prevent the flow becoming turbulent. The throughput can be increased by integrating multiple flow focusing channels in the same device, as in the case of NP and microparticle synthesis.

## Future perspectives

High cost and process variability hinder the translation of laboratory-scale technology into product commercialization. To comply with cGMP, technologies that offer reproducible and scalable production of biologically relevant materials must be developed. Microfluidics has emerged as a potential platform to advance biomanufacturing in the field of drug/gene/cell therapies via improved synthesis of nanoscale drug/gene delivery system, microencapsulation of drug/cells, intracellular delivery of macromolecules and fabrication of macroscale construct of micro-/nano-fibers. Microfluidics not only can improve the quality of drug/gene/cell delivery systems, it can also help establish precise structure-function relationships of NP and understand the intracellular delivery barriers. As nanotherapeutics become more sophisticated, requiring the integration of therapeutic, imaging and targeting modalities into the same NP, a reproducible fabrication process such as that afforded by microfluidic synthesis becomes even more important. As optimization of stem cell niche becomes more complex and requires precise patterning of physical and biochemical cues, microfluidics-assisted fabrication of biofunctional scaffolds can also play a more prominent role. There is no question that microfluidics can enhance the quality of drug/gene/cell delivery systems. The challenge is scaling-up these microfluidic technologies. Perhaps one can draw inspiration from the advance of computer science and engineering, where massively parallel processing systems have led to computational power capable of dealing with big data. One would think that the scale-up challenges highlighted in this perspective are solvable. At least that might be the case for precision medicine, where the scale of individualized therapeutic products would be addressable by microfluidic technologies.

As biomaterials innovations in the past decades have led to exciting conceptual advances in sophisticated device design, one of the grand challenges of biomaterials research in the 21st century has to be biomanufacturing. To date, translation of biomaterials innovations has been inadequate and under-appreciated. To address this deficiency, academia-industry collaboration, funding priority and innovation program establishment must be supported and reinforced. In parallel, training will be paramount. Innovations cannot be sustained without training, from the student to the professional level. Students should be taught principles such as automation, micro/nanofabrication, interface of physics and biology for biomaterials design and manufacturing principles. To facilitate this training, professors and industrial scientists should spend time in each other’s domains to learn the respective principles and practices. In essence, the field of biomaterials needs a new model for partnering industry and academia in the 21st century so as to increase the rate of translation for benefiting the society.
